# Observations on Abdominal Pressure in Obstetrical Practice

**Published:** 1819-04

**Authors:** W. Marson

**Affiliations:** Member of the Royal College of Surgeons.


*For the London Medical and Physical Journal.*

*Observations on Abdominal Pressure in Obstetrical Practice;* by W. Marson, Esq. Member of the Royal College of Surgeons.

I HAVE read with much attention your report of Dr. Clarke's Registry, No. 237, p. 401; and also the paper of Dr. Hamilton, No. 240, p. 121, on the importance of abdominal pressure in obstetrical practice; and with the greater pleasure, because I have so amply treated on the subject myself, in your Journal, fifteen years since, (vol. x. p. 433, and vol. xi. p. 328.) Dr. Hamilton, your correspondent, by referring to these papers, will give me credit for having been more illustrative on abdominal pressure than himself,—to the junior practitioner in particular; and he may not think his own time unprofitably spent in the perusal of them. It is true I have not carried abdominal pressure to the extent "of pursuing the fundus uteri in its contraction until the fœtus be expelled," believing that I have given it all the latitude I ought to do; and I mean every respect to Dr. Clarke and Dr. Hamilton, though I declare myself not very sanguine in the use of the pressure they speak of, nor am I an advocate for abdominal pressure by towels or bandages, which Dr. Hamilton alludes to.

I shall wave further remark, as the only motive of this paper is to put in a full view the importance of abdominal pressure in obstetrical practice, and to establish my claim of first bringing into notice what has recently been, I will say, supported by Dr. Clarke and Dr. Hamilton.

*Worksop, Notts; Feb.* 24, 1819.

